# Re-evaluation of Microhardness of Mineral Trioxide Aggregate and Biodentine After Removal From Root Using Different Types of Solvents: An In Vitro Study

**DOI:** 10.7759/cureus.69906

**Published:** 2024-09-22

**Authors:** Ruchali S Bhandare, Sudha B Mattigatti

**Affiliations:** 1 Department of Conservative Dentistry and Endodontics, School of Dental Science, Krishna Vishwa Vidyapeeth, Karad, IND

**Keywords:** biodentine, biomimetic materials, endodontic therapy, mta, pulp necrosis, retreatment, root canal therapy

## Abstract

Objective

This study aimed to evaluate the effects of different solvents on the microhardness of mineral trioxide aggregate (MTA) and Biodentine, which are used in endodontic therapy for their biocompatibility and ability to create a hermetic seal.

Methods

A total of 150 extracted human single-rooted teeth were selected and sectioned into 4 mm slices. The samples were divided into two groups according to the material used: MTA and Biodentine. Then the samples were incubated at 37°C and 100% humidity for 28 days. After that, 15 MTA and Biodentine samples each were sent for microhardness test and the remaining samples were subdivided into four subgroups according to the solvent used, i.e., treated with normal saline, 5.25% sodium hypochlorite, 10% citric acid, or 40% hydrofluoric acid. The microhardness of the upper and lower surfaces of the samples was measured using a Vickers microhardness tester.

Results

The average microhardness values for Biodentine and MTA were found to be 74.4 HV and 69.9 HV, respectively. Treatment with sodium hypochlorite, citric acid, and hydrofluoric acid significantly reduced the microhardness of both materials. Hydrofluoric acid had the most pronounced effect, followed by citric acid and sodium hypochlorite. Statistical analysis using one-way analysis of variance (ANOVA) 'F' test showed highly significant differences between the groups.

Discussion

The study demonstrated that solvents such as hydrofluoric acid, citric acid, and sodium hypochlorite effectively reduced the microhardness of MTA and Biodentine, facilitating their removal during retreatment. This reduction in microhardness is attributed to the chemical interactions of the solvents with the materials, leading to disintegration and reduced structural integrity.

Conclusion

Biodentine had greater microhardness compared to MTA, and hydroflouric acid reduced the microhardness to a greater extent compared to other solvents.

## Introduction

Changes in the pulp and peri-radicular tissues may result from trauma to the teeth’s calcified structures and their supporting tissues due to harmful stimuli. Bacterial, chemical, or physical stimuli can cause damage, with severe cases potentially leading to irreversible inflammation, pulp necrosis, and subsequent pathological changes in the surrounding tissues [1]. The debridement step in endodontic therapy involves the disruption and removal of this microbial ecosystem. The goal of nonsurgical endodontic therapy is to eliminate irritants from the canal and achieve a fluid-tight, three-dimensional obturation [2].

In certain challenging cases, such as vital pulp therapy-direct pulp capping, pulpotomy, root-end filling, apexification, and apexogenesis, it is impossible to achieve a three-dimensional hermetic seal by using gutta-percha. In these situations, biomimetic materials, such as Biodentine and mineral trioxide aggregate (MTA) are the preferred options. These materials enhance patient outcomes and potentially alter the prognosis of cases that were previously considered untreatable [3].

Despite these advances, many endodontic and restorative treatments continue to yield undesirable outcomes, largely due to the physical and chemical properties of the materials used. Even calcium silicate materials, such as MTA and Biodentine, have certain drawbacks, including the risk of tooth discoloration, high cost, extended setting times, handling difficulties, and challenges in retrieval after setting [4-11]. These limitations can complicate retreatment procedures because every root canal filling material must be retrievable in case of failure. Although root canal therapy generally has a high success rate, posttreatment failure is possible [12]. In such cases, rotary and ultrasonic instruments are often ineffective at removing MTA and Biodentine from the canal, and there is a risk of instrument separation. Therefore, a solvent is required for the retrieval of both Biodentine and MTA [13].

## Materials and methods

Materials

**Figure 1:**
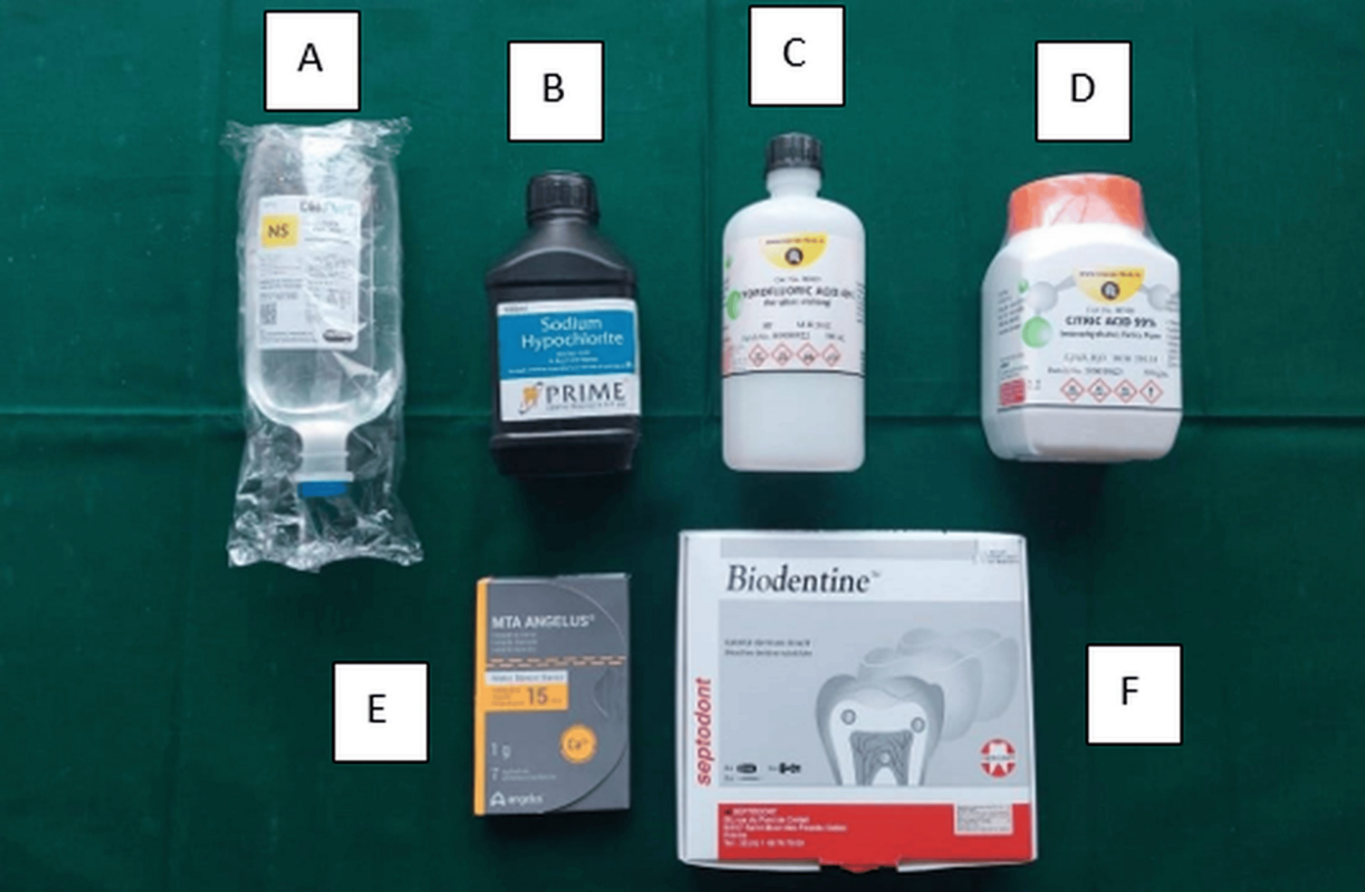
Materials used for dental procedure A. Normal saline B. 5.25% Sodium hypochlorite C. 40% Hydrofluoric acid D. 10% Citric acid E. White mineral trioxide aggregate (Angelous) F. Biodentine (Septodont)

The materials used in this study included normal saline, 5.25% sodium hypochlorite, 40% hydrofluoric acid, and 10% citric acid. In addition, white MTA (Angelus Odontologica, Londrina, Brazil) and Biodentine (Septodont, Saint-Maur-des-Fossés , France) were used as the primary dental materials. Figure 1 provides a visual representation of these materials.

Instruments

**Figure 2:**
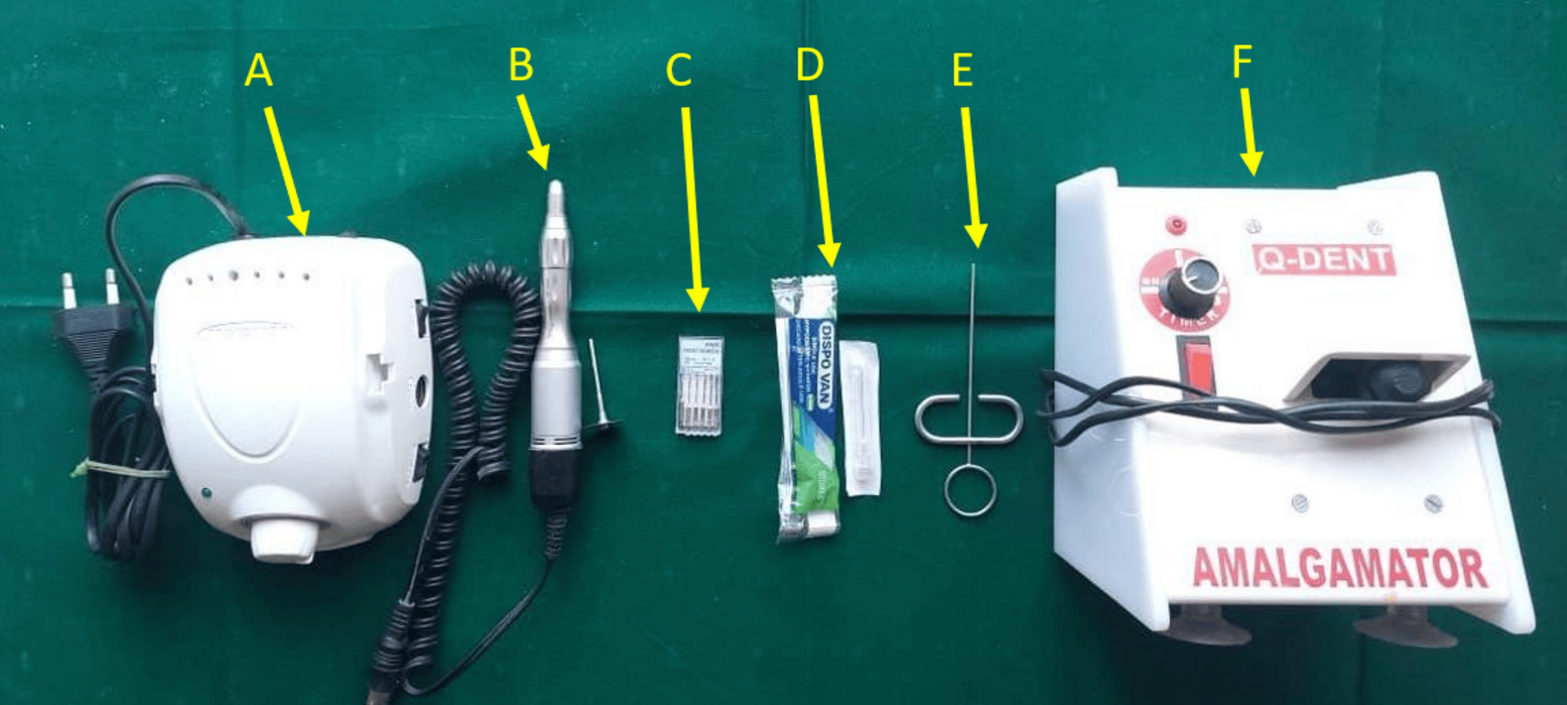
Instruments used for dental procedure A. Endodontic motor (Marathon M3) B. Straight micromotor handpiece and Diskbur (DD/D355-190-017F) C. # 1 to # 6 Gates glidden drills (Mani) D. 27-gauge needle E. Messing gun (Waldent) F. Amalgameter (Q-Dent)

The instruments used in this study included an endodontic motor (Marathon M3, Marathon Electric, Wausau, WI), a straight micromotor handpiece with a Diskbur (DD/D355-190-017F), Gates-Glidden drills (#1 to #6, Mani, Dentsply Sirona, Charlotte, NC), a 27-gauge needle, a messing gun (Waldent, New Delhi, India), and an amalgameter (Q-Dent, Denham Springs, LA). Figure 2 provides a visual representation of these instruments.

Equipment

The equipment used in this study included a universal testing machine and an incubator.

Selection of teeth

**Figure 3:**
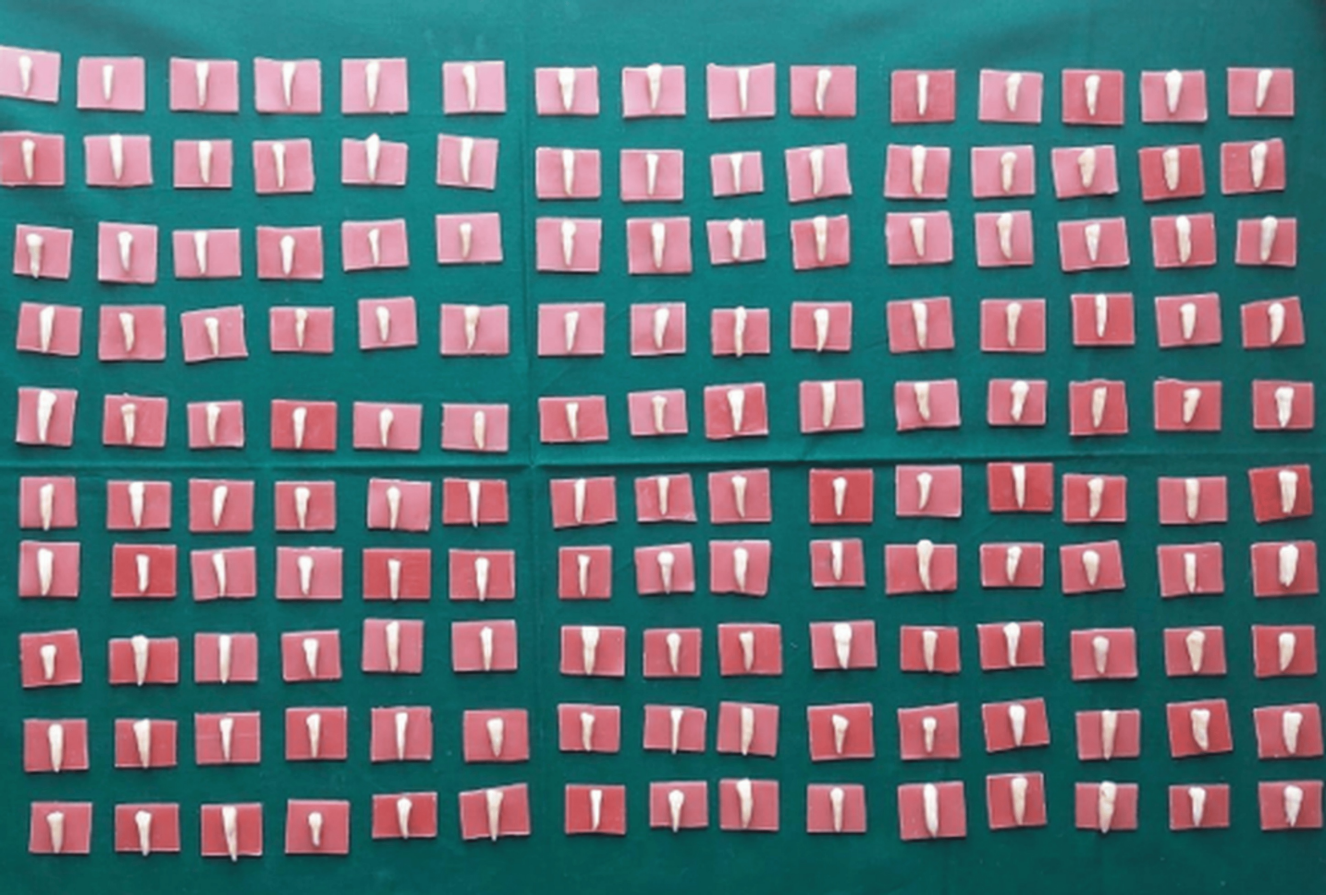
Extracted tooth samples

A total of 150 extracted human single-rooted teeth with a single root canal and apical foramen were selected using a convenient sampling method. Figure 3 presents the extracted tooth samples.

Standardization of samples

**Figure 4:**
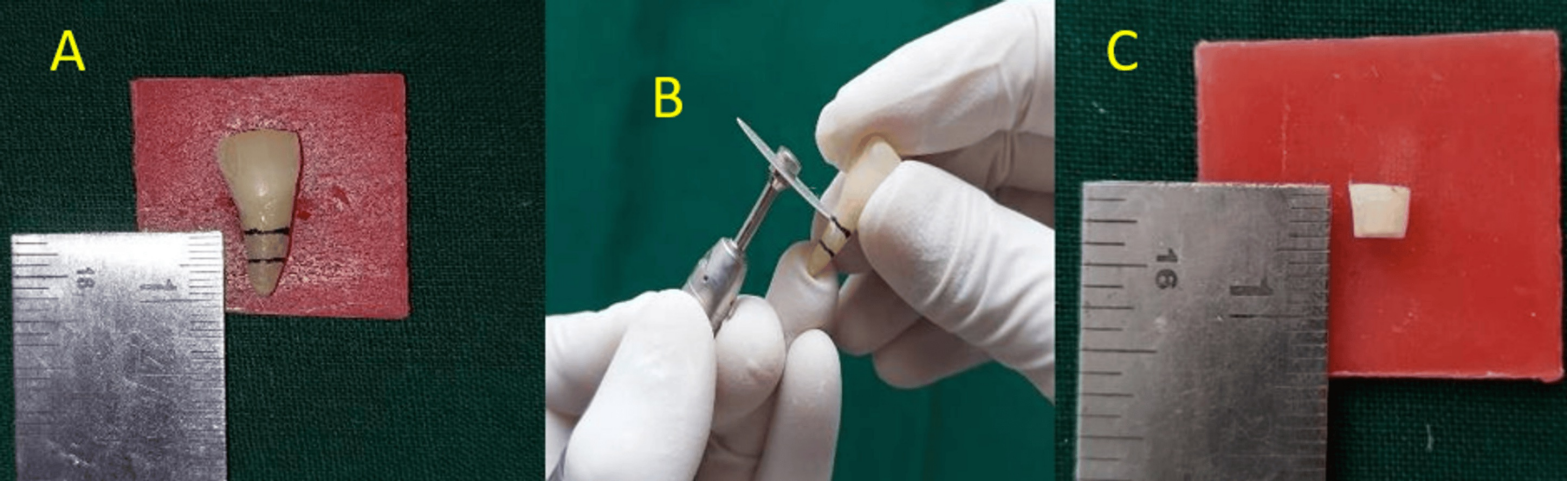
Standardization of samples A. Marking 4mm on root in the mid-root region B. Decoronation using a diamond disc and slow speed handpiece. C. Sectioned sample (4mm)

The extracted single-rooted teeth were standardised by first marking a 4 mm section at the mid-root region. Subsequently, the crowns were removed (decoronated) by using a diamond disc attached to a slow-speed handpiece. The teeth were then horizontally sectioned to obtain a 4-mm slice from the mid-root region. Figure 4 illustrates these steps, including the marking of the root, the decoronation process, and the final sectioned 4-mm sample.

**Figure 5:**
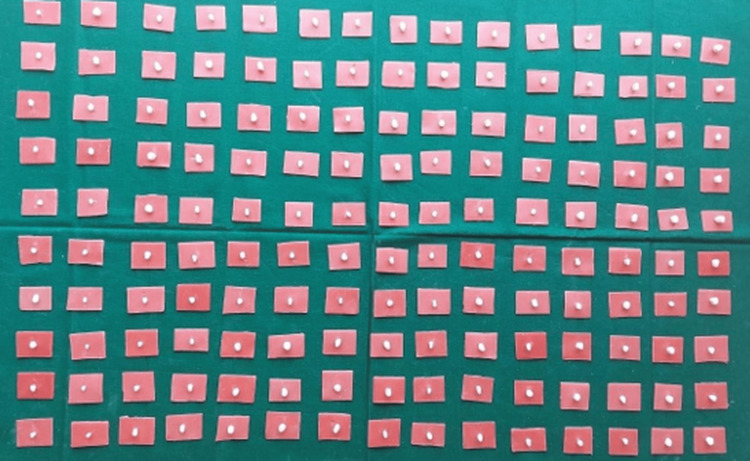
Teeth samples after sectioning

Figure 5 presents all the tooth samples after sectioning.

**Figure 6:**
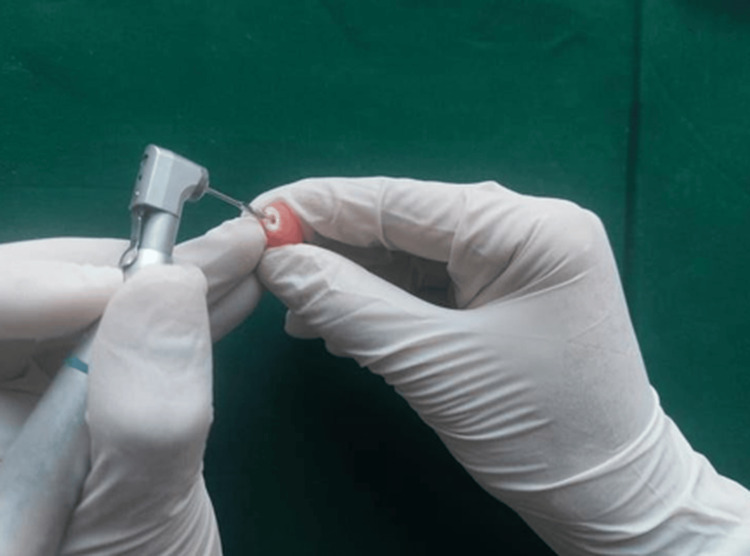
Space preparation with Gates-Glidden drills

Space preparation was performed using Gates-Glidden drills, as illustrated in Figure 6.

**Figure 7:**
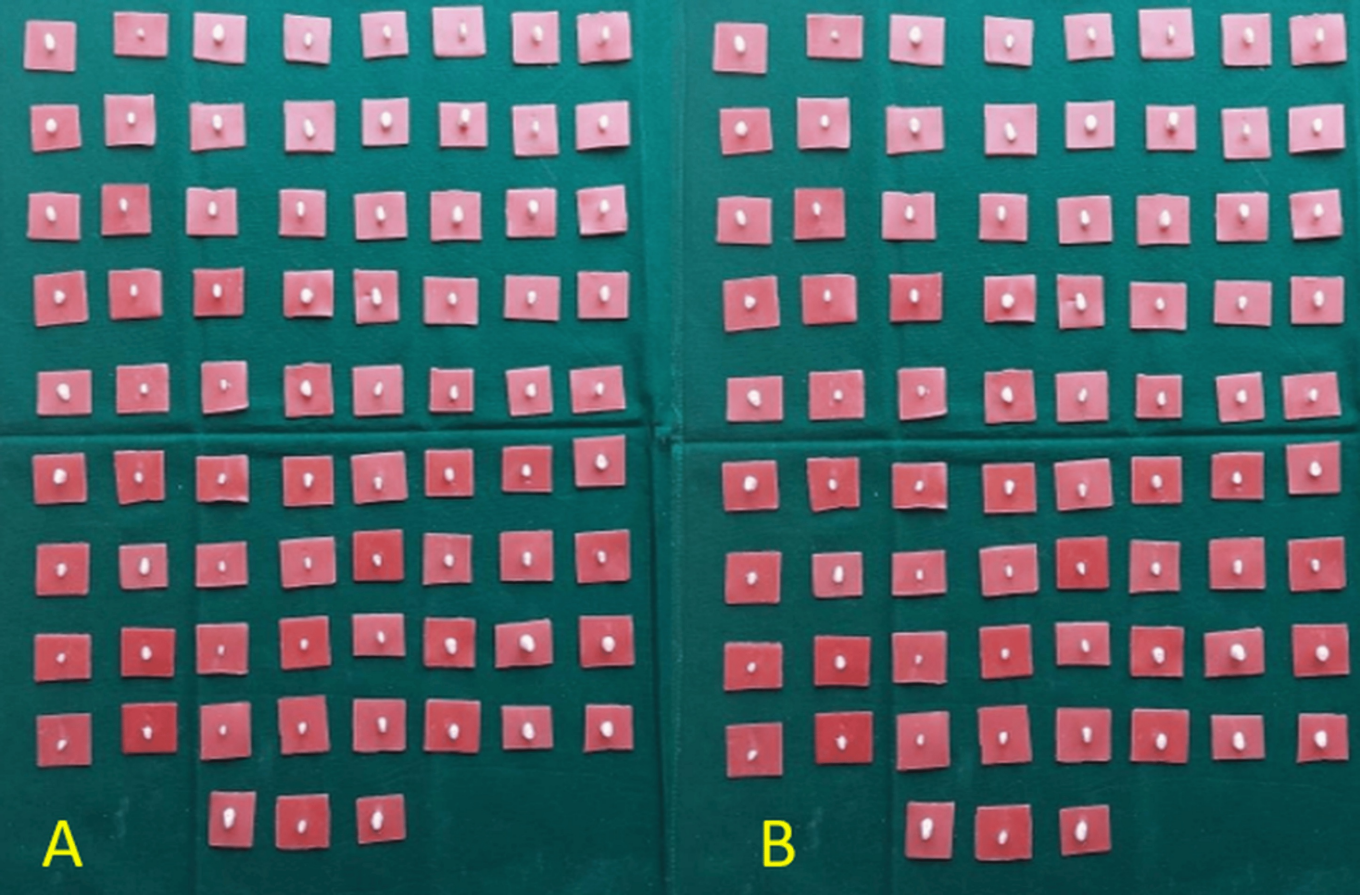
Sample size distribution A. 75 samples for mineral trioxide aggregate B. 75 samples for Biodentine

The study involved two groups: MTA and Biodentine. For each group, 75 samples were prepared, resulting in a total of 150 samples. Figure 7 illustrates all the samples prepared for both the MTA and Biodentine groups.

The manufacturer’s recommendations were followed for mixing MTA and Biodentine, which were then placed separately into the canal areas of each root slice. After removing any excess material by using a scalpel, the samples were incubated for 28 days at 37°C and 100% humidity.

The specimens were inspected under a microscope for cracks, defects, or material gaps. On day 28, 30 samples from each group were subjected to a Vickers microhardness test by using a universal testing machine to determine the microhardness of MTA and Biodentine.

The samples from the two main groups were further randomly divided into four subgroups. In the MTA group, the subgroups were as follows: Subgroup A (MTA and normal saline, n = 15), Subgroup B (MTA and 5.25% sodium hypochlorite, n = 15), Subgroup C (MTA and 10% citric acid, n = 15), and Subgroup D (MTA and 40% hydrofluoric acid, n = 15).

In the Biodentine group, the subgroups were as follows: Subgroup A (Biodentine and normal saline, n = 15), Subgroup B (Biodentine and 5.25% sodium hypochlorite, n = 15), Subgroup C (Biodentine and 10% citric acid, n = 15), and Subgroup D (Biodentine and 40% hydrofluoric acid, n = 15).

Two drops of the respective solutions were added to the samples: sodium hypochlorite to Subgroup A, citric acid to Subgroup B, and hydrofluoric acid to Subgroup C. Normal saline was added to Subgroup D, which served as the control group. After the solvent was added, the 4-mm specimens were divided into two 2-mm specimens each. On day 28, a Vickers microhardness test was conducted on both the upper and lower surfaces of each 2-mm specimen by using a universal testing machine to evaluate the microhardness of the various subgroup specimens.

## Results

The microhardness of Biodentine was tested on both the upper and lower surfaces by using a Vickers microhardness test. The average value was found to be approximately 74.4 HV on both surfaces (Table 1).

**Table 1:** Microhardness of Biodentine samples

Biodentine samples		
S. No.	Upper	Lower
1	74.8	73.8
2	74.5	74.5
3	73.9	74.9
4	74.6	74.8
5	73.8	73.9
6	74.6	73.8
7	74.7	73.9
8	73.9	74.7
9	74.5	74.4
10	74.4	74.5
11	74.3	74.8
12	74.8	74.6
13	73.8	74.3
14	74.9	74.2
15	74.4	74.8

The microhardness of MTA samples was tested on both the upper and lower surfaces, yielding an average value of approximately 69.9 HV on both surfaces (Table 2).

**Table 2:** Microhardness of MTA samples MTA: Mineral trioxide aggregate.

MTA samples		
S. No.	Upper	Lower
1	69.8	70.3
2	69.9	70.1
3	70.1	69.4
4	69.8	69.5
5	69.4	69.6
6	69.5	70.2
7	70.2	70.5
8	70.3	70.3
9	69.7	69.1
10	69.5	69.2
11	69.3	69.5
12	70.5	69.7
13	70.4	70.2
14	70.2	70.7
15	69.9	70.3

The microhardness of Biodentine samples in the control group in which saline was added was measured. We found average values of 74.3 HV on the upper surface (U-upper), 74.4 HV on the lower surface (U-lower), 74.4 HV on the upper surface (L-upper), and 74.5 HV on the lower surface (L-lower). Detailed results are presented in Table 3.

**Table 3:** Microhardness of Biodentine samples using saline as control group

Biodentine + Saline				
	Upper (U)		Lower (L)	
S. No.	U-Upper	U-Lower	L-Upper	L-Lower
1	74.8	74.5	74.8	74.8
2	74.5	74.8	74.7	74.7
3	74.6	74.9	74.2	74.1
4	74.5	74.1	74.1	74.9
5	74.8	74.3	74.3	74.3
6	74.2	74.8	73.8	73.8
7	74.1	74.5	73.9	73.9
8	73.2	73.8	73.7	74.1
9	73.4	73.9	74.8	74.2
10	73.9	73.7	74.9	74. 8
11	73.8	73.9	74.7	74.7
12	73.9	73.7	74.8	74.6
13	74.7	74.5	74.9	74.7
14	74.5	74.6	73.8	74.8
15	74.3	74.7	73.7	74.4

The microhardness of MTA samples in the control group in which saline was added was measured. On average, the values were 69.9 HV on the upper surface (U-upper), 70 HV on the lower surface (U-lower), 70.1 HV on the upper surface (L-upper), and 70.2 HV on the lower surface (L-lower). Detailed results are presented in Table 4.

**Table 4:** Microhardness of MTA after using saline as control group MTA: Mineral trioxide aggregate

MTA + Saline				
	Upper (U)		Lower (L)	
S. No.	U-Upper	U-Lower	L-Upper	L-Lower
1	69.8	70.2	69.8	69.9
2	69.2	70.3	69.2	70.1
3	70.1	69.9	70.2	70.5
4	70.5	69.8	70.6	70.6
5	70.6	69.7	70.7	70.3
6	69.8	70.5	69.9	70.4
7	69.9	70.6	70.1	69.8
8	70.4	70.2	70.2	69.8
9	70.2	70.1	70.3	69.9
10	70.1	69.8	69.8	70.6
11	70.5	69.9	70.5	70.7
12	69.8	70.7	70.6	70.5
13	69.9	70.3	70.3	70.2
14	68.8	69.7	70.2	70.3
15	69.9	69.6	69.8	69.8

The microhardness of Biodentine samples in which sodium hypochlorite was added as a solvent was measured. The average values were as follows: 64 HV on the upper surface (U-upper), 67.1 HV on the lower surface (U-lower), 67.5 HV on the upper surface (L-upper), and 67.9 HV on the lower surface (L-lower). Detailed results are presented in Table 5.

**Table 5:** Microhardness of Biodentine samples using sodium hypochlorite as a solvent NaOCl: sodium hypochlorite

Biodentine + NaOCl				
	Upper (U)		Lower (L)	
S. No.	U-Upper	U-Lower	L-Upper	L-Lower
1	64.8	68.8	67.8	68.4
2	63.5	66.5	68.1	66.8
3	63.9	65.9	66.8	67.3
4	63.8	66.3	66.7	68.2
5	64.7	67.8	68.2	68.5
6	64.4	66.8	68.5	68.3
7	63.8	67.2	68.3	68.8
8	63.5	67.5	68.2	68.5
9	63.4	67.3	66.8	68.3
10	64.4	67.2	67.2	68.9
11	64.3	66.5	67.8	67.8
12	64.3	66.3	67.2	67.9
13	64.2	67.8	67.9	67.5
14	63.9	67.7	66.8	67.2
15	63.8	67.3	66.7	67.3

The microhardness of MTA samples to which sodium hypochlorite was added as a solvent was measured. The average values were as follows: 63.9 HV on the upper surface (U-upper), 66.1 HV on the lower surface (U-lower), 66 HV on the upper surface (L-upper), and 65.7 HV on the lower surface (L-lower). Detailed results are presented in Table 6.

**Table 6:** Microhardness of MTA after using Sodium hypochlorite as solvent MTA - Mineral Trioxide Aggregate NaOCl - Sodium Hypochlorite

MTA + NaOCl				
	Upper (U)		Lower (L)	
S. No.	U-Upper	U-Lower	L-Upper	L-Lower
1	63.8	65.8	66.8	65.1
2	64.1	66.1	65.1	66.5
3	63.9	66.9	66.1	66.3
4	63.8	65.9	66.2	66.5
5	63.2	66.8	66.5	66.3
6	64.2	66.5	66.3	66.3
7	64.3	65.8	65.8	65.3
8	64.5	65.7	65.5	65.4
9	63.8	65.9	65.3	65.3
10	63.6	66.4	66.3	65.2
11	64.8	66.5	66.8	66.3
12	64.5	66.3	65.7	66.4
13	64.3	65.5	65.6	65.2
14	63.3	65.8	66.5	65.3
15	63.5	66.7	66.2	65.5

The microhardness of Biodentine samples to which citric acid was added as a solvent was measured. The average values were as follows: 62 HV on the upper surface (U-upper), 64.9 HV on the lower surface (U-lower), 65 HV on the upper surface (L-upper), and 64.9 HV on the lower surface (L-lower). Detailed results are presented in Table 7.

**Table 7:** Microhardness of Biodentine samples using citric acid as solvent CA: Citric acid

Biodentine + CA				
	Upper (U)		Lower (L)	
S. No.	U-Upper	U-Lower	L-Upper	L-Lower
1	62.1	64.8	65.5	65.6
2	61.2	65.6	64.8	64.4
3	61.8	64.1	64.9	65.5
4	62.3	65.5	65.2	65.5
5	62.4	64.4	65.3	65.3
6	61.8	65.7	64.7	65.8
7	61.7	64.8	64.6	64.2
8	61.6	64.8	64.4	64.8
9	62.8	64.9	65.8	64.2
10	62.7	65.6	65.7	64.8
11	62.1	65.1	65.6	64.1
12	62.8	65.2	65.3	64.2
13	61.8	65.3	65.4	65.1
14	61.7	64.4	64.3	65.8
15	61.6	64.5	64.8	65.5

The microhardness of MTA to which citric acid was added as a solvent was measured. The average values were as follows: 60.6 HV on the upper surface (U-upper), 63.9 HV on the lower surface (U-lower), 63.9 HV on the upper surface (L-upper), and 64.1 HV on the lower surface (L-lower). Detailed results are presented in Table 8.

**Table 8:** Microhardness of MTA after using citric acid as solvent MTA: Mineral trioxide aggregate; CA: citric acid

MTA + CA				
	Upper (U)		Lower (L)	
S. No.	U-Upper	U-Lower	L-Upper	L-Lower
1	60.2	63.1	63.2	64.6
2	61.3	64.5	64.3	64.6
3	60.6	63.8	64.1	64.5
4	59.8	64.5	63.5	64.4
5	60.1	63.8	63.2	63.3
6	60.2	63.7	64.4	63.8
7	60.5	63.3	64.3	63.7
8	61.5	63.2	63.8	64.2
9	61.3	64.5	63.6	64.1
10	61.2	64.4	64.2	64.8
11	60.3	64.6	64.8	64.7
12	60.2	63.8	64.2	64.6
13	60.5	63.7	63.9	63.3
14	60.3	64.1	63.8	63.4
15	61.8	64.2	63.7	64.4

The microhardness of Biodentine samples to which hydrofluoric acid was added as a solvent was measured. On average, the values were 58.4 HV on the upper surface (U-upper), 62 HV on the lower surface (U-lower), 62 HV on the upper surface (L-upper), and 61.8 HV on the lower surface (L-lower). Detailed results are presented in Table 9.

**Table 9:** Microhardness of Biodentine after using HF acid as solvent HF: Hydroflouric acid

Biodentine + Hydroflouric acid				
	Upper (U)		Lower (L)	
S. No.	U-Upper	U-Lower	L-Upper	L-Lower
1	60.5	62.8	62.8	61.1
2	59.8	61.6	61.6	60.6
3	59.5	63.3	63.3	60.9
4	58.3	61.5	62.5	61.8
5	58.4	61.3	62.8	61.5
6	57.3	61.2	61.2	62.8
7	57.8	62.8	61.8	62.5
8	57.3	62.7	61.5	62.7
9	57.8	62.5	61.3	62.5
10	58.1	61.1	62.5	61.8
11	58.4	62.2	62.3	61.5
12	57.7	62.8	62.8	61.3
13	57.7	61.8	61.1	61.8
14	57.8	61.5	61.8	62.2
15	60.2	62.2	61.5	62.8

The microhardness of MTA samples to which hydrofluoric acid was added as a solvent was measured. The average values on each surface were as follows: 57.9 HV on the upper surface (U-upper), 60.8 HV on the lower surface (U-lower), 61 HV on the upper surface (L-upper), and 60.8 HV on the lower surface (L-lower). Detailed results are presented in Table 10.

**Table 10:** Microhardness of MTA after using HF acid as solvent MTA: Mineral trioxide aggregate; HF: hydroflouric acid

MTA + Hydroflouric acid				
	Upper (U)		Lower (L)	
S. No.	U-Upper	U-Lower	L-Upper	L-Lower
1	57.3	60.6	61.1	61.3
2	58.2	61.5	60.2	60.3
3	57.8	60.3	60.5	60.2
4	58.9	60.2	61.8	60.1
5	57.2	60.4	60.8	60.5
6	57.8	60.5	60.5	60.3
7	57.2	61.2	61.2	61.3
8	57.7	61.8	61.5	61.5
9	58.1	61.6	61.3	61.8
10	58.8	61.7	60.7	61.5
11	58.3	60.5	60.9	60.8
12	58.5	60.3	60.8	60.7
13	58.4	60.4	61.5	60.8
14	57.9	60.9	61.3	60.5
15	57.8	61.5	61.8	61.1

The microhardness of MTA samples was compared after the application of two drops of sodium hypochlorite, citric acid, hydrofluoric acid, and saline (as a control group) on the upper and lower surfaces of 2-mm samples. The mean values for microhardness in Subgroup A (saline) were 69.96 HV on the U-upper surface, 70.08 HV on the U-lower surface, 70.14 HV on the L-upper surface, and 70.22 HV on the L-lower surface. In Subgroup B (sodium hypochlorite), the mean values were 63.97 HV on the U-upper surface, 66.17 HV on the U-lower surface, 66.04 HV on the L-upper surface, and 65.79 HV on the L-lower surface. For Subgroup C (citric acid), the mean values were 60.65 HV on the U-upper surface, 63.94 HV on the U-lower surface, 63.93 HV on the L-upper surface, and 64.16 HV on the L-lower surface. In Subgroup D (hydrofluoric acid), the mean values for microhardness were 57.99 HV on the U-upper surface, 60.89 HV on the U-lower surface, 61.06 HV on the L-upper surface, and 60.84 HV on the L-lower surface.

An overall comparative statistical analysis of MTA's microhardness to which sodium hypochlorite, citric acid, hydrofluoric acid, and saline were added as solvents was conducted using a one-way analysis of variance (ANOVA) ‘F’ test, which revealed a highly statistically significant difference (p < 0.001) among the groups. The data are presented in Table 11.

**Table 11:** Intragroup comparison of microhardness in Group 1 (MTA) after usage of different solvents p>0.05: not significant; *p<0.05: significant; **p<0.001: highly significant. MTA: Mineral trioxide aggregate; HF: hydroflouric acid; ANOVA: analysis of variance

Group 1 (MTA)	U-Upper Mean (SD)	U-Lower Mean (SD)	L-Upper Mean (SD)	L – Lower Mean (SD)
Subgroup A (Saline)	69.96 (0.48)	70.08 (0.34)	70.14 (0.39)	70.22 (0.32)
Subgroup B (Sodium hypochlorite)	63.97 (0.46)	66.17 (0.44)	66.04 (0.52)	65.79 (0.57)
Subgroup C (Citric acid)	60.65 (0.6)	63.94 (0.5)	63.93 (0.45)	64.16 (0.52)
Subgroup D (HF acid)	57.99 (0.53)	60.89 (0.58)	61.06 (0.48)	60.84 (0.53)
One way ANOVA F test value	F = 1451.0	F =981.0	F =1005.0	F =912.01
p value (overall), Significance	p<0.001**	p<0.001**	p<0.001**	p<0.001**
Subgroup A vs Subgroup B^	p<0.001**	p<0.001**	p<0.001**	p<0.001**
Subgroup A vs Subgroup C^	p<0.001**	p<0.001**	p<0.001**	p<0.001**
Subgroup A vs Subgroup D^	p<0.001**	p<0.001**	p<0.001**	p<0.001**
Subgroup B vs Subgroup C^	p<0.001**	p<0.001**	p<0.001**	p<0.001**
Subgroup B vs Subgroup D^	p<0.001**	p<0.001**	p<0.001**	p<0.001**
Subgroup C vs Subgroup D^	p<0.001**	p<0.001**	p<0.001**	p<0.001**

The microhardness of Biodentine samples was compared after the application of two drops of sodium hypochlorite, citric acid, hydrofluoric acid, and saline (as a control group) on the upper and lower surfaces of 2-mm samples. The mean values for microhardness in Subgroup A (Saline) were 74.21 HV on the U-upper surface, 74.31 HV on the U-lower surface, 74.34 HV on the L-upper surface, and 74.4 HV on the L-lower surface. In Subgroup B (sodium hypochlorite), the mean values were 64.04 HV on the U-upper surface, 67.12 HV on the U-lower surface, 67.53 HV on the L-upper surface, and 67.98 HV on the L-lower surface. For Subgroup C (citric acid), the mean values for microhardness were 62.02 HV on the U-upper surface, 64.98 HV on the U-lower surface, 65.08 HV on the L-upper surface, and 64.98 HV on the L-lower surface. In Subgroup D (hydrofluoric acid), the mean values were 74.4 HV on the U-upper surface, 58.44 HV on the U-lower surface, 62.05 HV on the L-upper surface, and 61.85 HV on the L-lower surface.

An overall comparative statistical analysis of the hardness of the Biodentine samples to which sodium hypochlorite, citric acid, hydrofluoric acid, and saline were added was conducted using a one-way ANOVA ‘F’ test, which revealed a highly statistically significant difference (p < 0.001) among the groups, as shown in Table 12.

**Table 12:** Intragroup comparison of microhardness in Group 2 (Biodentine) after usage of different solvents HF: Hydroflouric acid; ANOVA: analysis of variance

Group 2 (Biodentine)	U-Upper Mean (SD)	U-Lower Mean (SD)	L-Upper Mean (SD)	L – Lower Mean (SD)
Subgroup A (Saline)	74.21	74.31 (0.42)	74.34 (0.47)	74.4 (0.36)
Subgroup B (Sodium hypochlorite)	64.04	67.12 (0.75)	67.53 (0.66)	67.98 (0.63)
Subgroup C (Citric acid)	62.02 (0.48)	64.98 (0.5)	65.08 (0.47)	64.98 (0.63)
Subgroup D (HF acid)	74.4 (0.36)	58.44 (1.04)	62.05 (0.69)	61.85 (0.71)
One-way ANOVA F test value	F = 1556.0	F = 1087.0	F =1180.0	F = 1185.0
p value (overall), Significance	p<0.001**	p<0.001**	p<0.001**	p<0.001**
Subgroup A vs Subgroup B^	p<0.001**	p<0.001**	p<0.001**	p<0.001**
Subgroup A vs Subgroup C^	p<0.001**	p<0.001**	p<0.001**	p<0.001**
Subgroup A vs Subgroup D^	p<0.001**	p<0.001**	p<0.001**	p<0.001**
Subgroup B vs Subgroup C^	p<0.001**	p<0.001**	p<0.001**	p<0.001**
Subgroup B vs Subgroup D^	p<0.001**	p<0.001**	p<0.001**	p<0.001**
Subgroup C vs Subgroup D^	p<0.001**	p<0.001**	p<0.001**	p<0.001**

A comparison of microhardness between Group 1 (MTA) and Group 2 (Biodentine) after treatment with saline in Subgroup A revealed a statistically significant difference (p < 0.001) between the two groups. Similarly, when comparing the microhardness of MTA and Biodentine samples in Subgroup B (sodium hypochlorite), the difference on the U-upper surface was not statistically significant (p > 0.05), whereas a statistically significant difference (p < 0.001) was observed on the L-upper, U-lower, and L-lower surfaces (Table 13).

**Table 13:** Intergroup comparison of microhardness between Group 1 (MTA) and Group 2 (Biodentine) after usage in Subgroup A and B MTA: Mineral trioxide aggregate

Subgroup A (Saline)	U-Upper Mean (SD)	U-Lower Mean (SD)	L-Upper Mean (SD)	L – Lower Mean (SD)
Group 1 (MTA)	69.96 (0.48)	70.08 (0.34)	70.14 (0.39)	70.22 (0.32)
Group 2 (Biodentine)	74.21	74.31 (0.42)	74.34 (0.47)	74.4 (0.36)
Unpaired t test	t = -23.792	t = -29.694	t = -26.118	t = -33.046
P value, Significance	p<0.001**	p<0.001**	p<0.001**	p<0.001**
Subgroup B (Sodium hypochlorite)	U-Upper Mean (SD)	U-Lower Mean (SD)	L-Upper Mean (SD)	L – Lower Mean (SD)
Group 1 (MTA)	63.97 (0.46)	66.17 (0.44)	66.04 (0.52)	65.79 (0.57)
Group 2 (Biodentine)	64.04	67.12 (0.75)	67.53 (0.66)	67.98 (0.63)
Unpaired t test	t = -0.446	t = -4.216	t = -6.782	t = -9.906
P value, Significance	P =0.659	p<0.001**	p<0.001**	p<0.001**

A comparison of microhardness between Group 1 (MTA) and Group 2 (Biodentine) after treatment with citric acid in Subgroup C revealed a statistically significant difference (p < 0.001) between the two groups on the U-upper, L-upper, and U-lower surfaces and a significant difference (p < 0.005) on the L-lower surface. Furthermore, a comparison between MTA and Biodentine samples in Subgroup D (hydrofluoric acid) showed that the difference on the U-upper surface was not statistically significant (p > 0.05); however, a statistically significant difference (p<0.001) was observed on the L-upper, U-lower, and L-lower surfaces, as shown in Table 14.

**Table 14:** Intergroup comparison of microhardness between Group 1 (MTA) and Group 2 (Biodentine) after usage in Subgroups C and D MTA: Mineral trioxide aggregate; HF: hydroflouric acid

Subgroup C (Citric acid)	U-Upper Mean (SD)	U-Lower Mean (SD)	L-Upper Mean (SD)	L – Lower Mean (SD)
Group 1 (MTA)	60.65 (0.6)	63.94 (0.5)	63.93 (0.45)	64.16 (0.52)
Group 2 (Biodentine)	62.02 (0.48)	64.98 (0.5)	65.08 (0.47)	64.98 (0.63)
Unpaired t-test	t = -6.864	t = -5.651	t = - 6.783	t = -3.872
P value, Significance	p<0.001**	P<0.001**	p< 0.001**	p =0.001*
Subgroup D (HF acid)	U-Upper Mean (SD)	U-Lower Mean (SD)	L-Upper Mean (SD)	L – Lower Mean (SD)
Group 1 (MTA)	57.99 (0.53)	60.89 (0.58)	61.06 (0.48)	60.84 (0.53)
Group 2 (Biodentine)	74.4 (0.36)	58.44 (1.04)	62.05 (0.69)	61.85 (0.71)
Unpaired t test	t = -1.471	t = -5.029	t = -4.526	t = -4.368
P value, Significance	p = 0.152	p<0.001**	p<0.001**	p<0.001**

## Discussion

During the debridement process known as endodontic therapy, the microbial ecosystem is disrupted and eliminated. For teeth with periapical disease, the goal of nonsurgical endodontic therapy is to achieve fluid-impervious obturation by biomechanically preparing the canal and removing any irritants [2].

Biomimetic materials, such as MTA and Biodentine, are recommended for certain challenging cases, such as in vital pulp therapy, including direct pulp capping, pulpotomy, root-end filling, apexification, and apexogenesis [3].

In this study, we focus on using three solvents - hydrofluoric acid, citric acid, and sodium hypochlorite - to reduce the microhardness of MTA and Biodentine. Torabinejad et al. introduced the cementitious material, commonly known as MTA, in 1993, which rapidly gained acceptance in dentistry [14]. Patented and marketed in 1995 [15], MTA was approved for endodontic applications in 1998 [16]. Two types of MTA are available: grey MTA (GMTA) and white MTA (WMTA). The main difference between GMTA and WMTA, as identified through electron probe microanalysis and scanning electron microscopy, is the concentrations of Al_2_O_3_ and MgO. A study published in 2006 reported that the initial setting times for GMTA and WMTA were approximately 2 h and 55 min and 2 h and 20 min, respectively. To overcome the lengthy setting time of MTA, the concentration of calcium sulphate was reduced, resulting in a final setting time of 15 min [17].

IIn 2009, Saghiri et al. investigated the physical properties of MTA and found that its compressive strength reached 40.0 MPa after 24 h and increased to 67.3 MPa after 21 days [18]. In 2010, Nandini et al. revealed that when set MTA is exposed to water, it releases calcium hydroxide, which may contribute to its ability to induce cementogenesis [19].

Another material of interest is Biodentine, often referred to as ‘dentine in a capsule’ and recognised as a ‘biocompatible and bioactive dentine substitute.’ Biodentine was developed to overcome the disadvantages associated with MTA and calcium hydroxide. In 2011, Priyalakshmi and Ranjan highlighted its unique properties. Biodentine consists of a fine, hydrophilic powder, similar to MTA powder, but is modified with softeners and setting accelerators. Biodentine is provided in a newly developed pre-dosed capsule formulation designed for use in a mixing apparatus. Upon application, Biodentine induces mineralisation through the formation of osteodentin, which is driven by early mineralisation, the expression of odontoblast markers, and increased TGF-β1 release from pulpal cells [20].

Guneser et al. demonstrated that Biodentine has a significantly higher push-out bond strength than MTA [21]. The higher dislodgement resistance of Biodentine than that of MTA is likely due to its biomineralisation ability, which may involve the formation of tags. The elastic modulus of Biodentine (22.0 GPa) is almost similar to that of dentin (18.5 GPa) [22]. The average compressive strength of dentin is 290 MPa, whereas that of Biodentine is 220 MPa. In addition, the microhardness of Biodentine is 60 HVN, which is comparable to that of natural dentin [22]. These mechanical characteristics make Biodentine an effective dentin substitute.

Despite these favourable properties, many endodontic and clinical restorative procedures still fail, often due to the physical and chemical characteristics of the materials used. In cases of failure, the retrieval of root canal filling materials becomes necessary to allow for potential retreatment [23]. Although root canal therapy generally has a high success rate, posttreatment failures may occur. In such situations, rotary and ultrasonic instruments are often ineffective in removing MTA and Biodentine from the canal, and there is a risk of instrument separation [11]. Therefore, a solvent is required to reduce the microhardness of both Biodentine and MTA to facilitate their removal [13].

The microhardness of materials can be reduced using solvents. In this study, the microhardness of MTA and Biodentine was reduced by adding the solvents hydrofluoric acid, citric acid, and sodium hypochlorite and then the microhardness of the materials was tested to assess deformation. Microhardness has an inverse relationship with porosity and is affected by various fundamental properties of materials, including tensile strength, modulus of elasticity, and the stability of their crystal structure [24]. Microhardness tests were performed as indicators of the setting process and to evaluate the quality and progression of the hydration process [19].

The microhardness test was conducted on day 28 because the material’s hydration process must be complete for the results to be valid. Torabinejad et al. reported that Biodentine sets in 12 min, whereas MTA requires 21 days to reach its maximum compressive strength. In addition, the complete chemical setting of MTA occurs in 21 days, whereas the complete chemical setting of Biodentine occurs requires a single day. This finding suggests that by day 28, both MTA and Biodentine would have fully hydrated, making it an appropriate time to assess their microhardness [17].

In the present study, MTA maturation and hydration reactions were found to extend significantly beyond the clinically observable setting time when MTA and Biodentine were mixed, and most of these reactions occurred during the first week of setting. When water reacted with tricalcium silicate and dicalcium silicate in MTA, the primary hydration products were calcium hydroxide (CH) and calcium silicate hydrate (C-S-H). The amount of CH in hydrated MTA increased from 4 h to one week after mixing and then gradually decreased after one month. At any given time, CH constituted approximately 10.8%, 1.9%, to 14.4% of the MTA mix. The C-S-H gel formed was poorly crystalline, and only CH crystals, known as ‘Portlandite’, were distinguishable through X-ray diffraction. Thus, the typical cubic or hexagonal Portlandite crystals with laminated or stratified structures were only recognisable in larger sizes within the hydrated MTA [6].

The setting reaction of Biodentine, which occurs between its powder and liquid components, led to the cement setting and hardening. Shortly after mixing, calcium silicate particles in Biodentine interacted with water, creating a high pH solution containing silicate ions, OH^−^, and Ca^2+^ ions. This reaction formed a hydrated calcium silicate gel on the cement particles and initiated the nucleation of calcium hydroxide as the tricalcium silicate underwent hydration. Over time, the hydrated calcium silicate gel polymerised, forming a solid network, whereas the release of calcium hydroxide ions increased the alkalinity of the surrounding medium. In addition, the hydrated calcium silicate gel enveloped the unreacted tricalcium silicate particles, and due to its water-impermeable nature, it slowed down further reactions [20].

The results of the current study indicated that low pH environments substantially affected the microstructure and physicochemical properties of MTA and Biodentine. Acidic conditions reduce microhardness by causing a selective loss of the matrix surrounding the crystalline structures on the surface of MTA and Biodentine. At a pH of 5, the cubic CH crystals in the hydrated samples become underdeveloped, with poorly defined crystal borders, and there is a noticeable decrease in the Portlandite peak in X-ray diffraction. CH is considered the weakest component in cement, making it more susceptible to chemical attack [6].

Yazdi et al. reported that exposure to an acidic pH inhibits CH crystal formation in MTA and Biodentine [25]. This finding aligns with the results of the current study, where the exposure to solvents led to decreased microhardness values. Similarly, Elnaghy reported changes in the microhardness of MTA and Biodentine at different pH values [26].

In the present study, hydrofluoric acid was found to more severely reduce the surface microhardness of MTA and Biodentine compared with citric acid and sodium hypochlorite. This effect is attributed to the fact that both the mechanical properties and the chemical structure of these materials degrade in acidic environments. Saghiri et al. concluded that both the type of acid and pH significantly affect Biodentine’s microhardness [18]. The penetration of root canal sealer into dentinal tubules can be affected by remnants of previous restorative materials, which may act as a physical barrier and promote adhesion. Various factors can affect the efficiency and completeness of MTA and Biodentine removal, including the type of manual or mechanical instruments used, irrigation methods, exposure to organic acids, and other procedural variables. The concentration and type of acids, the duration of exposure, chemical reactions between the acids and the materials, and the irrigation and instrumentation techniques employed can all affect the extent to which MTA and Biodentine can be retrieved.

In the current study, the microhardness of MTA and Biodentine decreased significantly after treatment with 40% hydrofluoric acid. Regardless of the material tested, the effects of hydrofluoric acid treatment were evident as increased surface roughness, characterised by irregularities and pores on the treated surfaces. This finding suggests that hydrofluoric acid induces dissolution, leading to a marked reduction in microhardness [27].

Dąbrowska et al. found that calcium ions dissolved to a great extent when MTA and Biodentine samples were irrigated with hydrofluoric acid and citric acid, particularly after ultrasonic activation [28]. The crystalline forms of MTA and Biodentine were extracted from the set cement matrix by using the acid, which can be attributed to the inhibition of calcium hydroxide crystal formation in acidic environments. In the current study, the reduced microhardness values observed when MTA and Biodentine were treated with 40% hydrofluoric acid can be explained by the disintegration of the material.

Citric acid produces a hydrosoluble chelate when it reacts with calcium silicate, making it easier to remove after ultrasonic activation [28]. This material disintegration phenomenon accounts for the decreased microhardness values. In addition, the compositions of Portland cement, MTA, and Biodentine are similar. Kastiukas et al. demonstrated a decrease in the strength of Portland cement with varying concentrations of citric acid. This finding is relevant to the current study because it highlights the similar effects of citric acid on related materials [29].

Sodium hypochlorite is the most widely accepted root canal irrigant and is commonly used in clinical practice at concentrations ranging from 0.5% to 6%. MTA sets by causing the hydration of Ca_2_SiO_4_, producing its hydrates and calcium hydroxide. However, Yamashita et al. demonstrated that sodium hypochlorite inhibits the synthesis of calcium hydroxide in MTA and that the release of Ca^2+^ ions can induce the formation of hard tissue as well as inhibit the formation of mineralised nodules [30].

When Biodentine comes into contact with dentinal tubules, it forms tag-like structures that act as micromechanical anchors. Upon reacting with sodium hypochlorite, these structures may lead to cohesive bond failure. This finding aligns with that of a previous study indicating that cohesive bonds are the primary cause of Biodentine-dentin bond failures [31].

The effectiveness of acids and chelating agents in decalcifying cement depends on several factors, including the application method, duration of exposure, pH, solution concentration, and hardness of the set cement [3]. In the current study, all the solvents used were found to significantly reduce the microhardness of the set cement.

## Conclusions

Although MTA and Biodentine are valuable materials in endodontic therapy, their retrievability can be enhanced using specific solvents that reduce their microhardness. This finding has crucial clinical implications, particularly in cases of retreatment. However, the choice of the solvent and its application must be carefully managed to balance the benefits of reduced microhardness with the potential risks of material degradation and weakening.

Within the limitations of this study, all three solvents - hydrofluoric acid, citric acid, and sodium hypochlorite - led to a significant reduction in the microhardness of both Biodentine and MTA. Practitioners should be aware of the effects of these solvents on the retrieval of MTA and Biodentine during retreatment. The critical task is the selection of an appropriate solvent, which is essential to effectively reduce microhardness without compromising the structural integrity of the materials.
